# Quantitative structural organization of the sclera in chicks after deprivation myopia measured with second harmonic generation microscopy

**DOI:** 10.3389/fmed.2024.1462024

**Published:** 2024-10-22

**Authors:** Juan M. Bueno, Rosa M. Martínez-Ojeda, Enrique J. Fernández, Marita Feldkaemper

**Affiliations:** ^1^Laboratorio de Óptica, Instituto Universitario de Investigación en Óptica y Nanofísica, Universidad de Murcia, Murcia, Spain; ^2^Section of Neurobiology of the Eye, Institute for Ophthalmic Research, Tuebingen, Germany

**Keywords:** myopia, deprivation, sclera, collagen, second harmonic microscopy

## Abstract

Visual deprivation causes enhanced eye growth and the development of myopia, which is associated with a change in the arrangement of collagen fibers within the sclera. A second harmonic generation (SHG) microscope has been used to image the collagen fibers of unstained scleral punches from the posterior part of chicken eyes. We aimed to analyze the fibrous scleral tissue and quantify the changes in collagen organization in relation to the extent of induced deprivation myopia. The scleral architecture was assessed with the Radon transform (RT) through the parameter called structural dispersion (SD) that provides an objective tool to quantify the level of organization of the collagen network. We found that final refraction and axial length changes were linearly correlated. However, no significant differences in scleral thickness were found for different amounts of induced myopia. In contrast, a significant correlation between SD and refraction was demonstrated, ranging from a non-organized (in the control sclerae) to a quasi-aligned distribution (with a dominant direction of the fibers, in the sclera of myopic chicks). These findings demonstrate a remodeling process of the scleral collagen associated with myopia progression that can be measured accurately combining SHG imaging microscopy and RT algorithms.

## 1 Introduction

The sclera is the white outer shell of the eye, a tough connective tissue with a complex organization of collagen fibers. It contains approximately 50% collagen by weight (about 90% type I) (1). This dense collagenous structure provides protection against external injury. Different microscopy techniques have been used to investigate the structure of the sclera and to measure the preferred orientations of the collagen lamellae. These include bright-field, scanning electron and atomic force microscopy, among others (2–5).

Myopia is an ocular condition resulting from a mismatch between the eye’s optical power and its axial length. The size and shape of the myopic eye are partly determined by the resistance of the sclera (6). Early studies of the human myopic eye detected thinner collagen fiber bundles and reduced scleral thickness at the posterior pole of the eye as compared with the emmetropic eye (6, 7). A study in monkeys’ eyes with experimentally induced myopia found that smaller fiber diameters are associated with a marked scleral thinning (8). Scleral thinning was also shown to occur during the development of axial myopia in a shrew model (9). Extensive details on the role of the sclera in the development of myopia can be seen in the work by McBrien and Gentle (10).

Chickens have been widely used as an animal model in myopia studies (11–13). The induction of visual deprivation using monocular diffusers or defocus with negative lenses has been reported to cause ocular elongation and subsequent myopia (11), which is associated with scleral growth (14) and increased creep rate of posterior and equatorial sclera (15). The chick’s sclera is composed of two layers: an outer fibrous layer (similar to that of the mammals), mainly composed of collagen type I and an inner cartilaginous layer, which contains collagen types II and IV, and aggrecan as the predominant cartilage proteoglycan (16, 17).

Second harmonic generation (SHG) microscopy is a non-lineal imaging modality especially suitable for visualizing collagen-based samples without the need of chemical markers, fixation procedures or histological preparation (18–20). Due to the rich collagen content of the sclera, this ocular component has been widely studied using this technique (21–26). Several experiments have used *ex-vivo* non-stained samples from both porcine (21, 22, 25) and human eyes (23, 24, 26). More recently, SHG images of the sclera in living human eyes have also been successfully obtained (27). However, to the best of our knowledge experiments combining SHG imaging and chicken scleras have not been previously reported.

It has been shown that the spatial arrangement of collagen in the sclera is altered by various conditions such as glaucoma (28), aging (29), or myopia (9, 10) to name a few. However, some previous studies on scleral changes were not fully consistent. More recently, quantitative analyses on the scleral arrangement as a function of myopia in guinea pig eyes using SHG imaging microscopy have been reported (30, 31). In particular, this article goes a step further on this topic and we evaluate and quantify the changes suffered by the sclera of myopic chickens by combining SHG images and the Radon transform (RT).

## 2 Materials and methods

### 2.1 Animals and tissue preparation

The scleral tissue used in this study was obtained from 13 chickens (aged 7–10 days) examined as part of a study on the development of myopia. All experiments were conducted in accordance with the ARVO statement for the use of animals and approved by the University of Tübingen Commission for Animal Welfare. White Leghorn chickens were raised under an 11/13 h light/dark cycle, with the light phase starting at 8:00 a.m. Water and food were supplied *ad libitum*. Illumination was provided by light bulbs that produced an average ambient illuminance of 500 lx on the cage floor.

Deprivation myopia was induced by attaching translucent plastic diffusers (32) over one eye for 7 days. Fellow eyes had normal vision and served as contralateral controls. Refractive state was measured without cycloplegia right before diffuser treatment started and at the end of the treatment period by automated infrared photoretinoscopy (33). Ocular dimensions were determined by A-scan ultrasonography as previously described (32), also at the beginning and at the end of the treatment period. Averages of three measurements for both refraction and axial length from contralateral control and treated eyes were taken.

**Figure 1:**
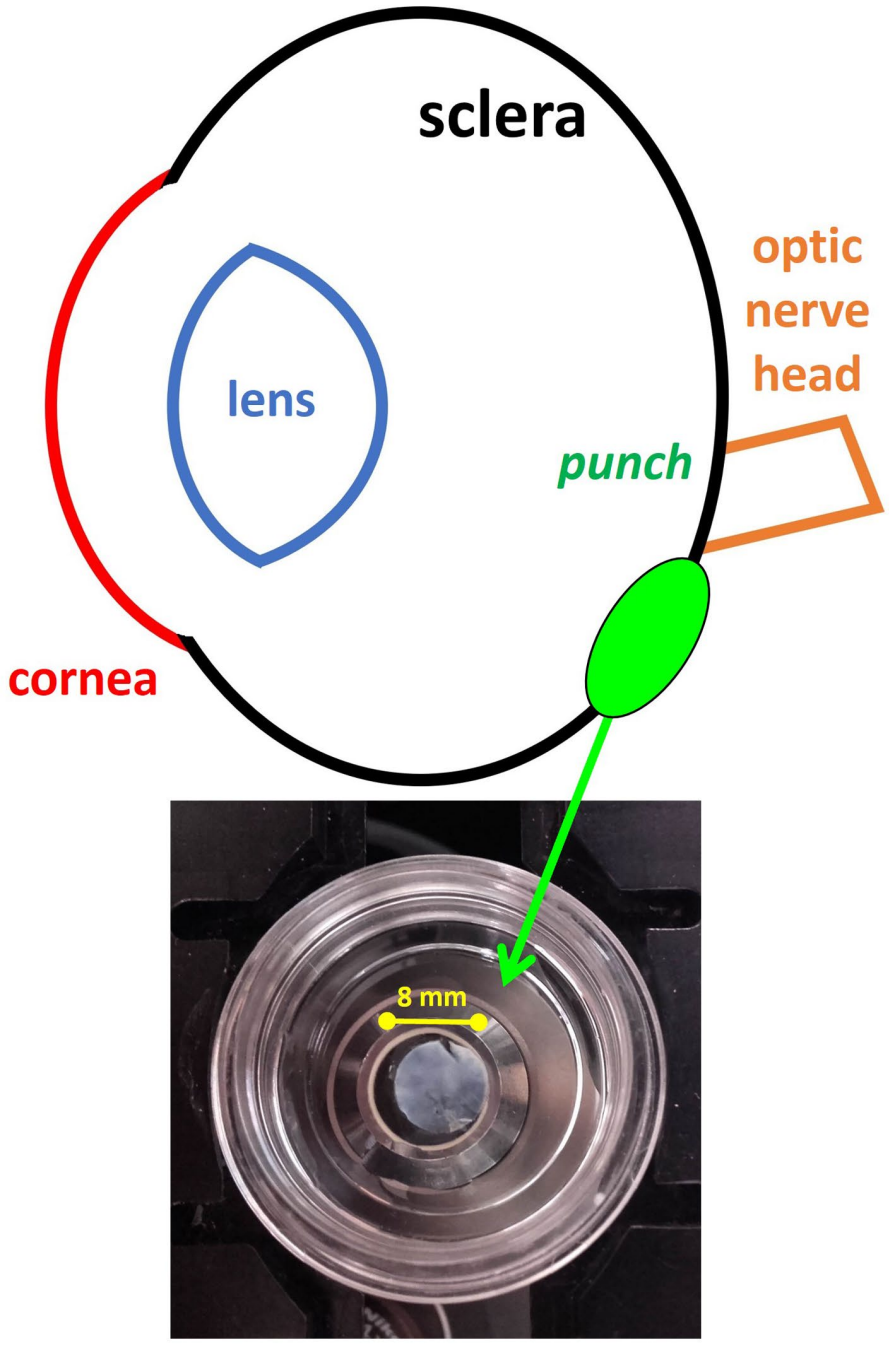
Illustrative drawing representing the location from where the chicken sclera tissue was harvested. The inset picture shows the scleral punch placed on the microscope glass bottom dish.

Animals were sacrificed by an overdose of ether. The eyes were immediately enucleated and cut with a razor blade in the equatorial plane, approximately 1 mm posterior to the ora serrata. The anterior segment of the eye was discarded and the vitreous removed. From each ocular globe, tissue punches (8-mm in diameter) were taken from an area close to the optic nerve head (Figure 1). Then, both the retinal pigment epithelium and the choroidal layers were removed. The remaining scleral tissue was fixed by 30 min of immersion in 4% paraformaldehyde (PFA) in 0.1 M phosphate buffer, washed and afterwards stored to be sent in 1% PFA solution for SHG imaging.

### 2.2 SHG image acquisition

SHG images of the sclera samples were obtained using a multiphoton microscopy system [see (34) for further details of the setup]. In brief, the instrument combined a commercial inverted microscope (TE2000-U; Nikon, Japan) with a 800-nm (central wavelength) Ti:sapphire laser (Mira900f; Coherent, St Clara, CA). The repetition frequency of the laser was 76 MHz, and the pulse width was ~120 fs. The beam was focused on the sample through a long working distance objective (20x, NA 0.50, Nikon, ELWD series), with an average power of 80 mW. The focusing objective collected the nonlinear signal emitted by the sample. The signal emitted in the backward direction passed through a dedicated narrow-band spectral filter (400 ± 10 nm) before reaching the detector. The detector was a photomultiplier tube (PMT; R7205-01, Hamamatsu). A DC motor coupled to the objective allowed optical sectioning across the entire specimen along the Z-direction.

Each non-stained scleral punch (see section 2.3) was placed on the microscope stage with its fibrous layer facing down on a glass bottom dish filled with phosphate buffer. The inset in Figure 1 shows one of the specimens prepared for SHG image acquisition.

**Figure 2:**
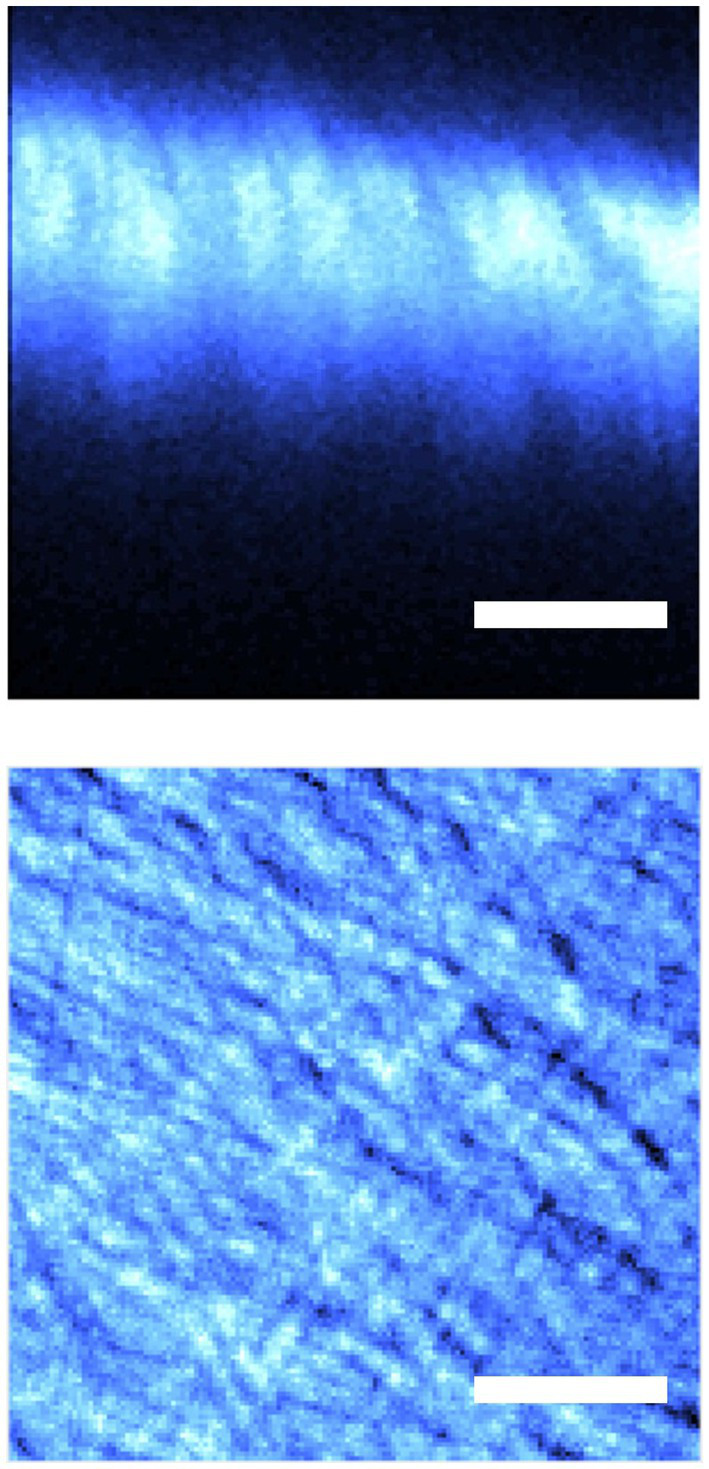
SHG images of the chicken sclera: axial tomography (top) and image across an XY plane (bottom). Scale bar: 50 μm.

Two imaging protocols were used to record the SHG signal from the scleral tissue: tomographic imaging (35) and “regular” XY-plane imaging. The latter was set to operate at 1 frame/s. SHG images were 180 × 180 μm^2^ in size (256 × 256 pixel^2^) and corresponded to the plane with best intensity projection within the sclera. For the former, the separation across adjacent points was 2 μm and it was used to image the fibrous portion of the chicken sclera. Whereas XY images allowed the visualization of the sclera collagen fibers, tomographic images permitted the calculation of the sample’s thickness (see section 2.3). No image averaging was performed. Illustrative examples of SHG images acquired with both protocols are presented in Figure 2.

### 2.3 Image analysis

**Figure 3:**
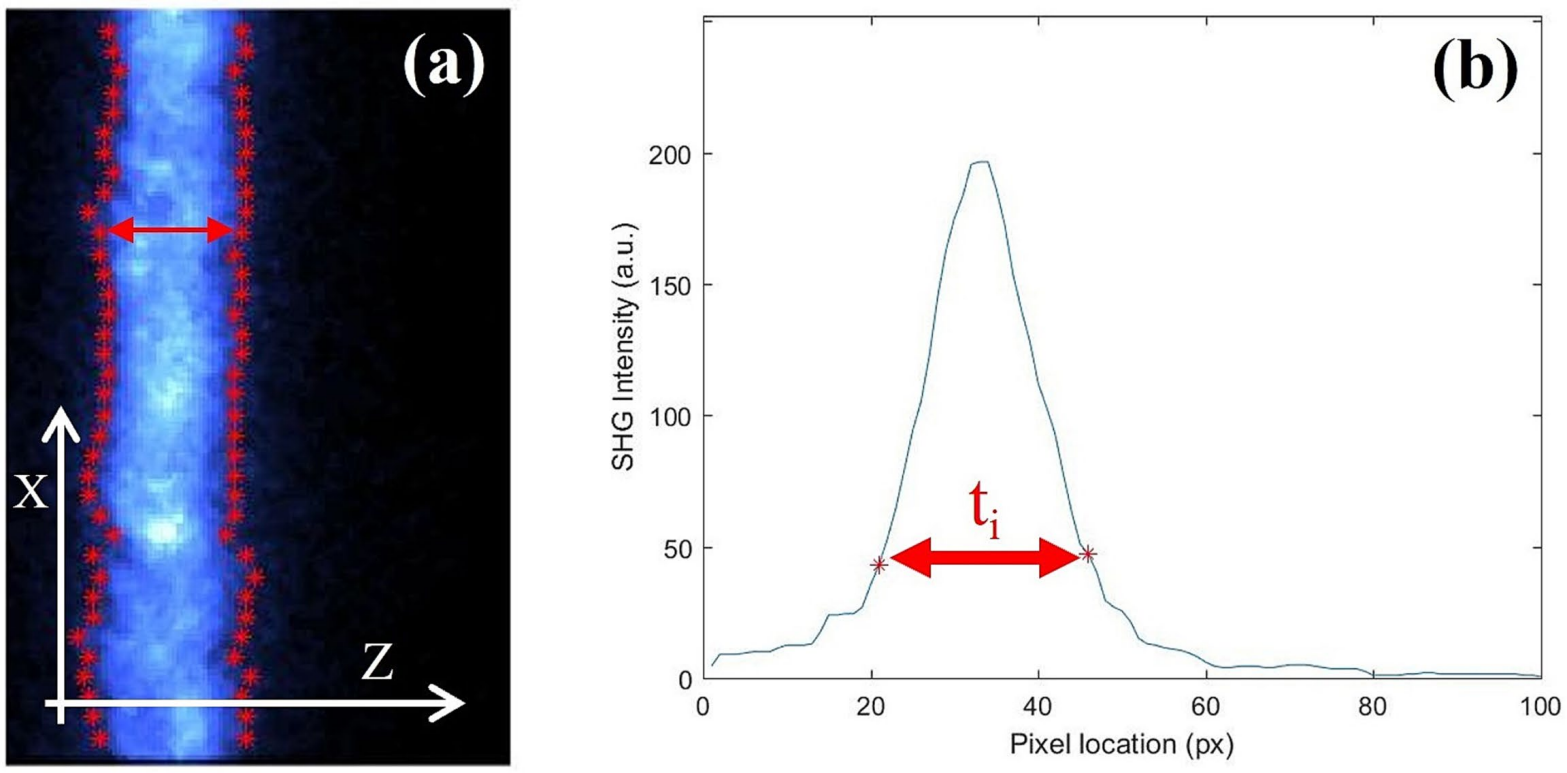
(a) Tomographic SHG images of a chicken sclera along the X direction. Red dots set the position of the edge estimated after the derivative of the curve at every point. (b) Example of an intensity profile as a function of depth for a particular X_i_ location.

Image processing was performed by a dedicated software developed under MatLab™ (The MathWorks, Inc., Natick, MA). Fibrous scleral thickness of the was obtained from the tomographic images as follows (Figure 3). For each X_i_ location (vertical axis on Figure 3a) the intensity profile along the Z axis was extracted (horizontal axis on Figures 3a,b). For each set of data forming an axial profile, the corresponding derivatives were used for an accurate edge detection. Then, the distance between every pair of edge points (at both sides of the profile) was taken as the local thickness (t_i_, see red arrows in Figure 3). The final thickness for each sample used herein was the mean value across all X_i_ locations.

**Figure 4:**
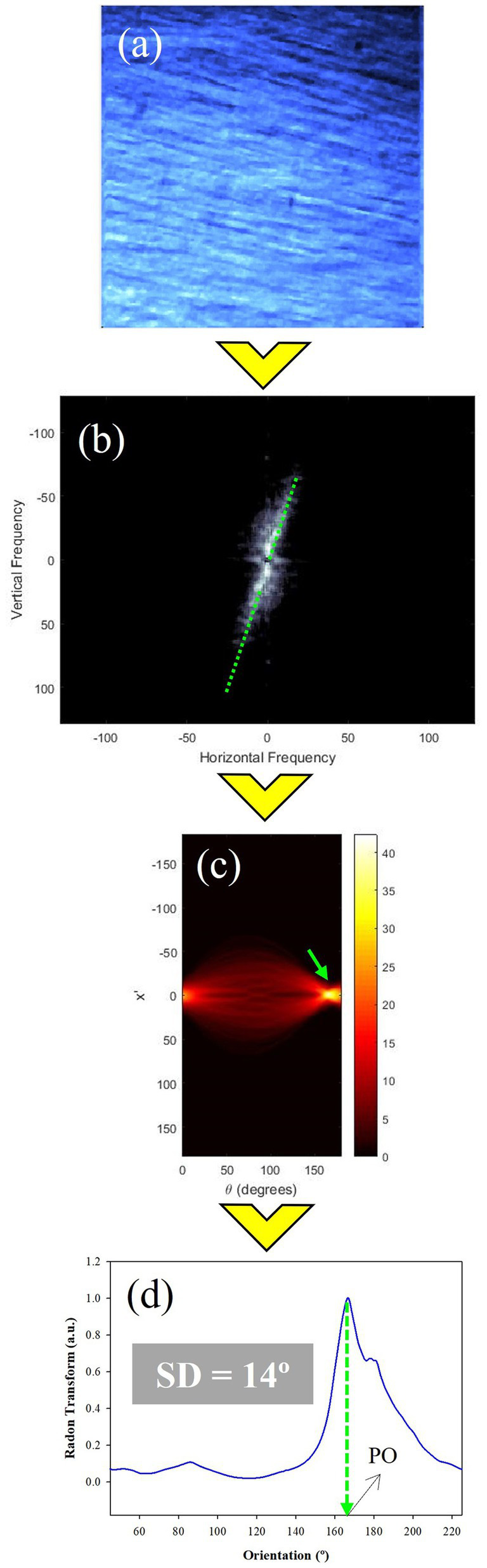
RT procedure to perform the calculation of the structural organization of the collagen fibers of the fibrous chicken sclera. (a) Original SHG image; (b) FFT image; (c) RT of the FFT image where the peak (green arrow) corresponds to the preferential orientation; (d) RT plot for x′ = 0. The insets indicate the values of SD and PO.

To explore the organization of the scleral collagen fibers of each SHG image (Figure 4a), the RT method was applied. This is a mathematical tool that combined with the Fourier Transform (FFT) robustly quantifies the preferential (or dominant) orientation (PO) of the fibers (Figures 4b,c) and the structural dispersion (SD) value. A detailed description of this formalism and its advantages when wavy collagen fibers and crimped architecture appear in the image can be found in (36). The RT of the FFT image ensures that all peaks are close to the center of the x′ reference axis (Figure 4c). This x′ = 0 line represents the angular information of the fibers (Figure 4d). In particular, the peak provides the PO (green arrow), and the SD is computed as standard deviation of the values of the angular distribution (37). If a PO exists, the distribution of Figure 4d can be fitted by a Gaussian function centered on the actual PO value. When SD ≤ 20°, the sample is composed of fibers quasi-aligned along a PO. For a sample presenting a non-organized structure, SD will be larger than 40°. A partially organized distribution is considered when SD values are within the range (20°, 40°]. As a general rule, the higher the SD, the lower the level of organization of the fibers within the tissue. At this step the algorithm is not designed to compute the thickness of the fibers with the SHG image.

### 2.4 Statistics

**Figure 5:**
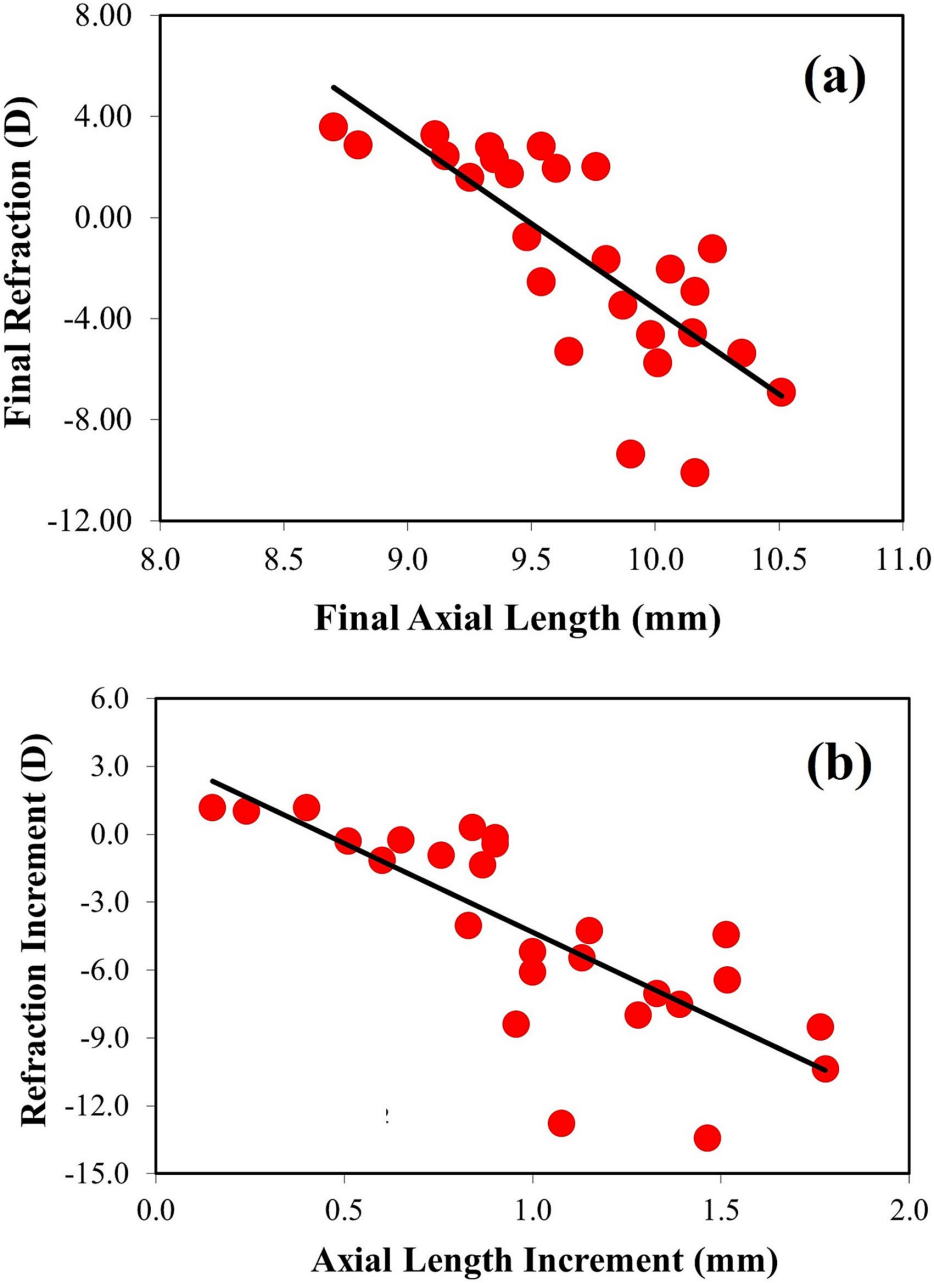
(a) Relationship between refraction (in D) and axial length (in mm) (best linear fit (solid black line): Rx_final_ = −6.74*AL_final_ + 63.74). (b) Change in refraction during the treatment period as a function of change in axial length (best linear fit (solid black line): ΔRx_final_ = 7.86*ΔAL_final_ + 3.52).

**Figure 6:**
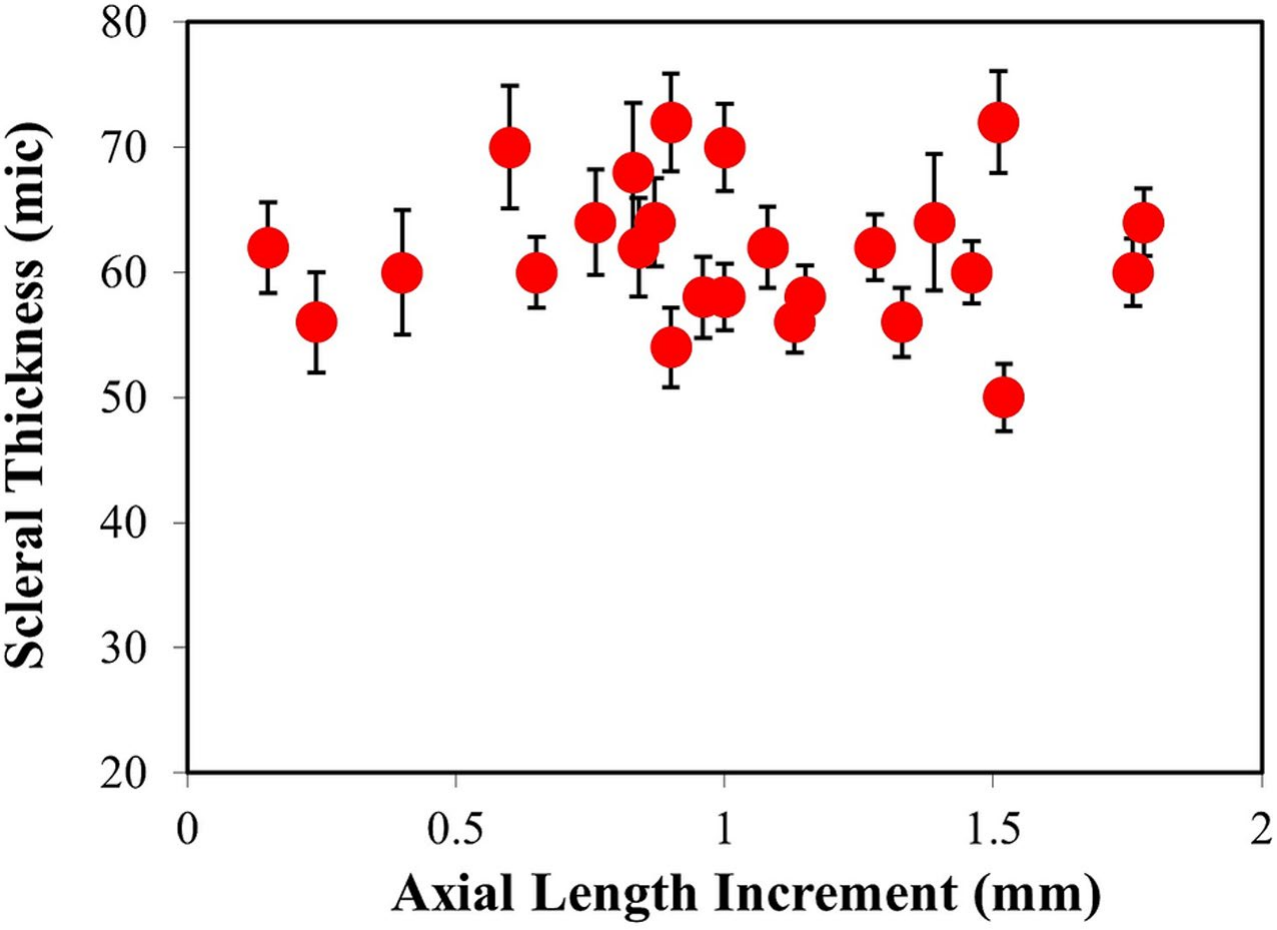
Averaged values of fibrous scleral thickness (μm) vs. the increment in axial length (mm).

**Figure 7:**
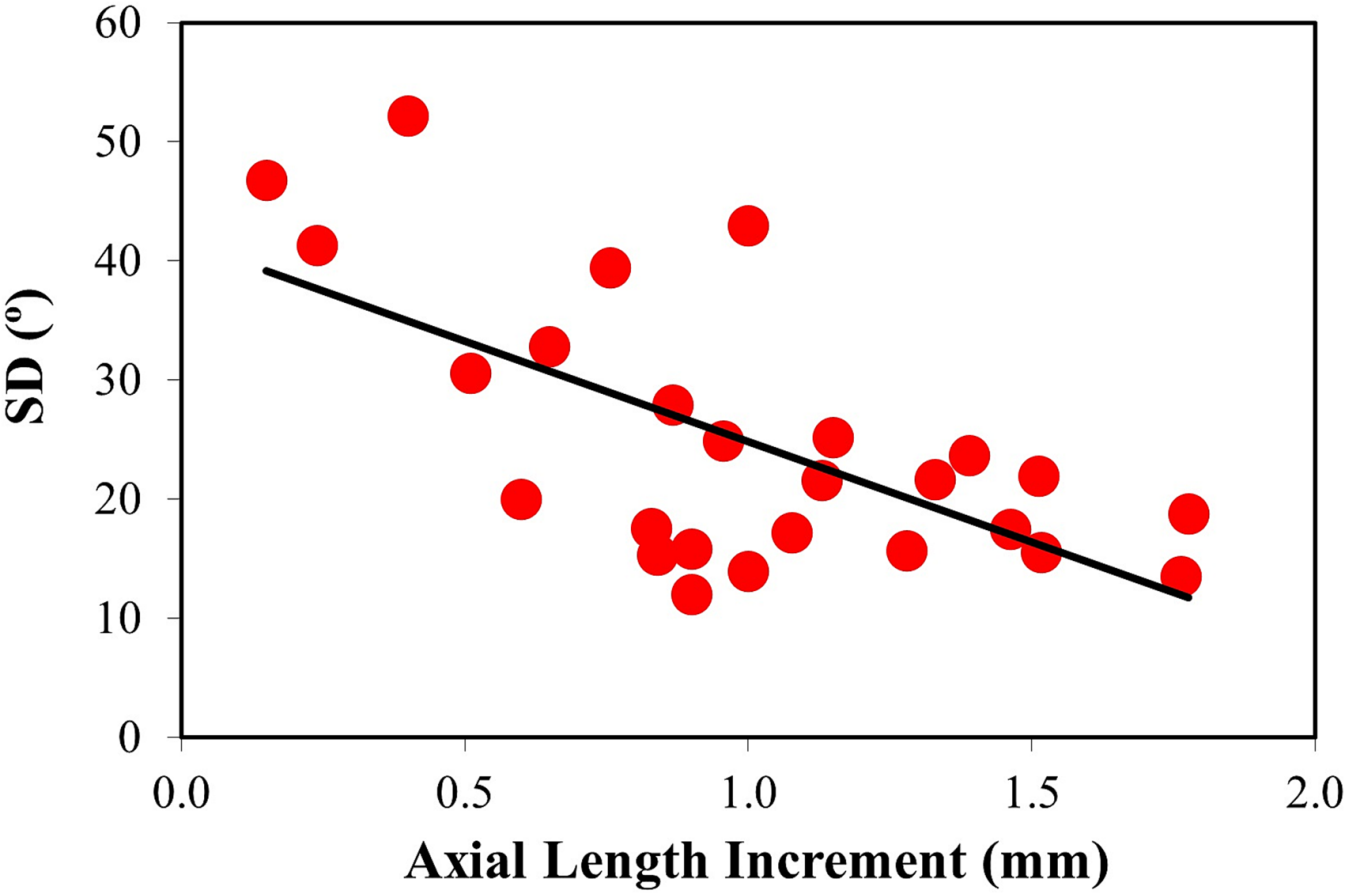
Changes in SD with axial length increment. Linear regression fitted to the data shows a statistically significant decrease. Best linear fit: SD = −16.85·ΔAL + 41.66.

Data are shown as the mean ± standard deviation. The difference between the experimental eyes and the fellow eyes were analyzed with a paired *t*-test. In Figures 5b, 6, 7, the measured parameters were expressed as changes/increment (*Δ*, after-before).

## 3 Results

### 3.1 Refraction and axial length

**Figure 8:**
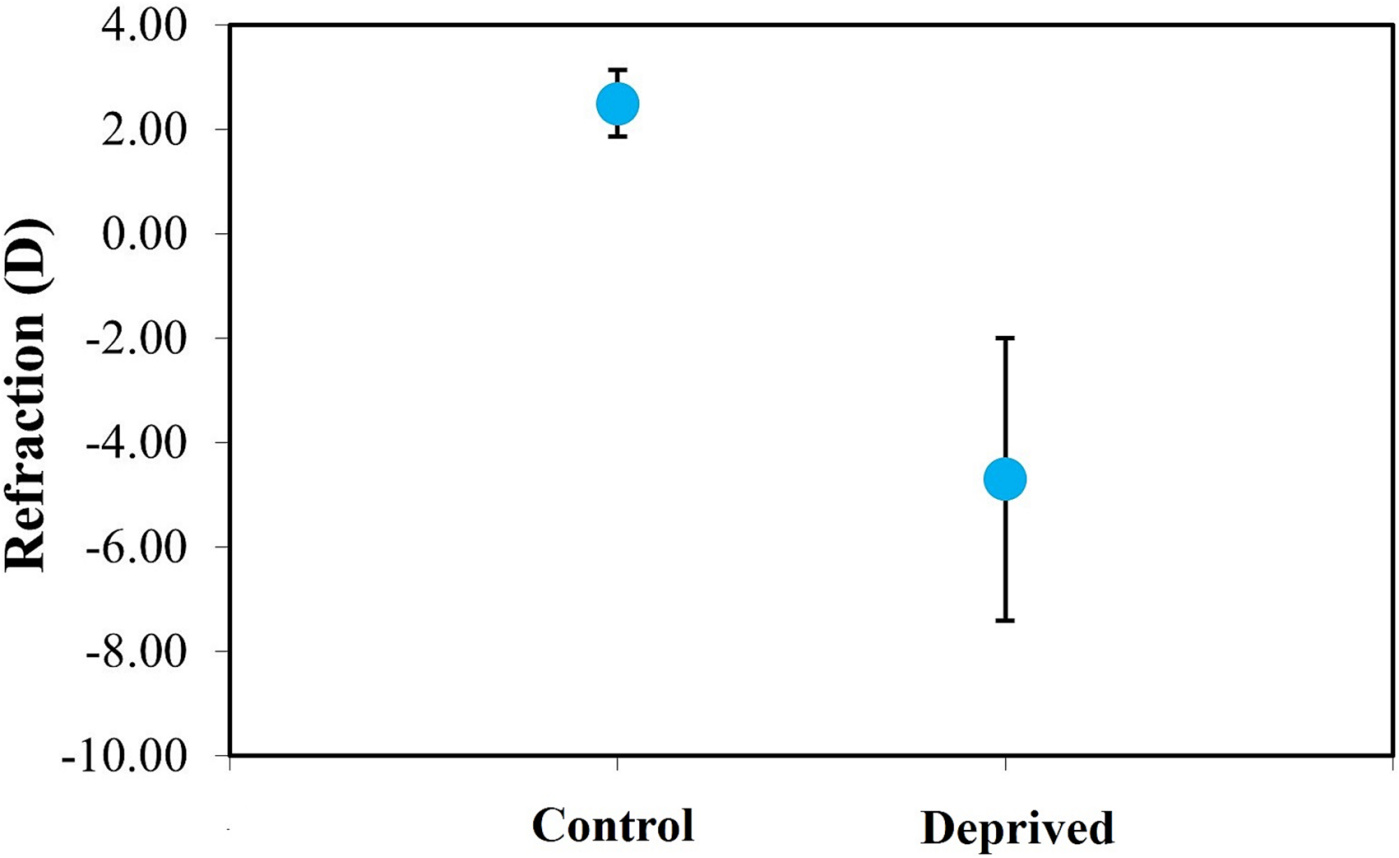
Average refraction in control and deprived chicken eyes (after 1 week of wearing a diffuser). Error bars indicate the standard deviation.

The diffuser-treated eyes developed axial myopia during the 1-week treatment period (mean final refraction: −4.70 ± 2.71 D, see Figure 8). Fellow control eyes, which were exposed to normal visual experience, remained slightly hyperopic (+2.49 ± 0.64 D). The refraction values between the two groups of eyes were statistically different (paired *t*-test, *p* < 0.0001).

Figure 5a shows the individual refraction as a function of the axial length after deprivation. It can be seen that higher degrees of myopia are closely associated with longer ocular length. This result is as expected. In the figure, the black line shows the best linear fit from the experimental data, which is significant (*R* = 0.77, *p* < 0.0001). The final refraction values were in the range of −10.1 D to +3.6 D. For completeness, the relationship between the change from baseline in refraction (ΔRx) and axial length (ΔAL) is shown in Figure 5b. Again, a statistically significant linear correlation was found (*R* = 0.78, *p* < 0.0001).

### 3.2 Fibrous scleral thickness

**Figure 9:**
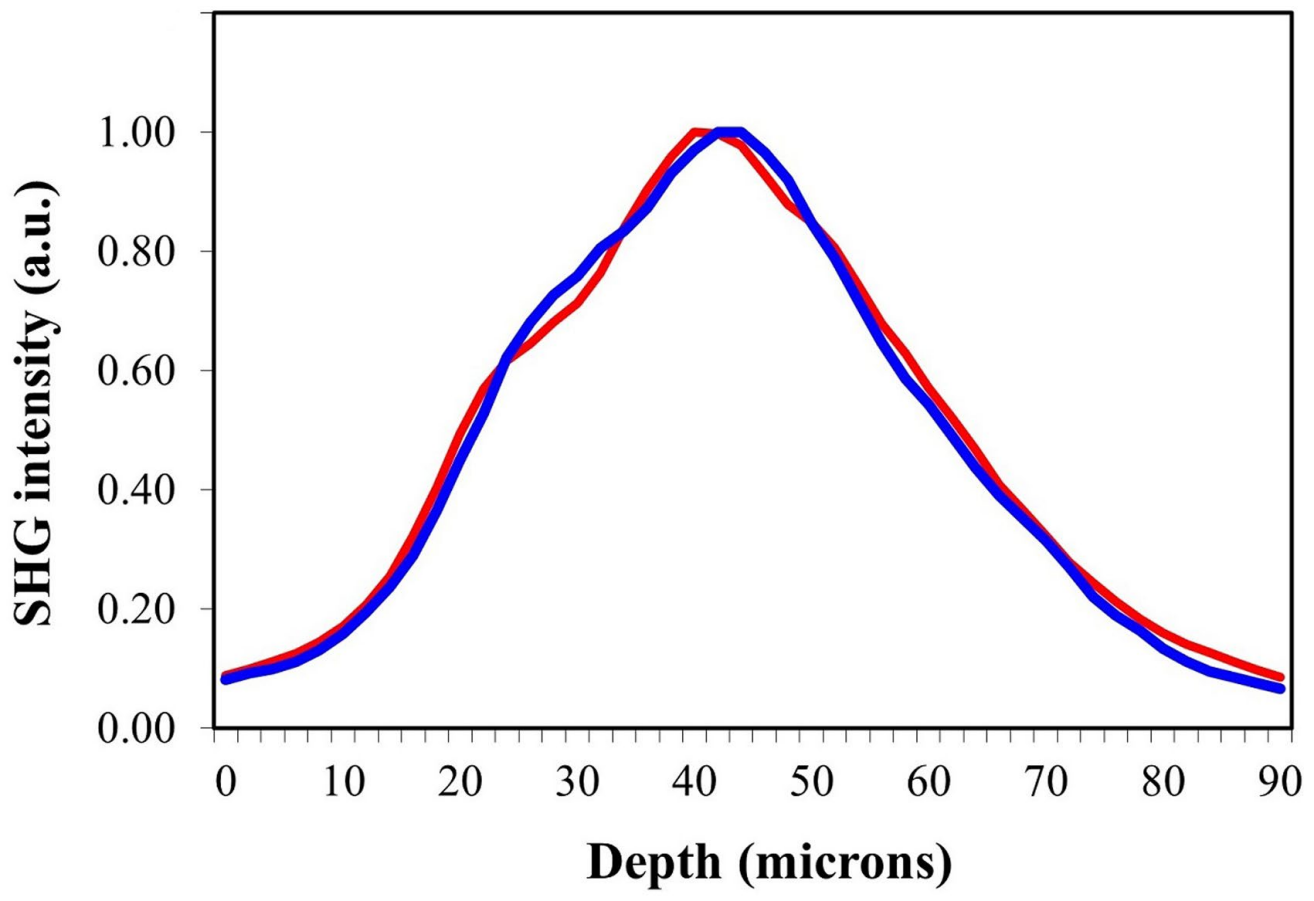
SHG intensity profiles as a function of depth for scleral punches from myopic eyes with refraction values of −1.23 D (blue) and − 9.35 D (red).

As an example, Figure 9 shows two randomly chosen SHG intensity profiles along the depth location (i.e., the Z-axis of the tissue as shown in Figure 3) for scleras from chicken eyes with low and high levels of myopia. Visual inspection reveals little difference between the two profiles, resulting in similar thickness values.

To confirm the apparent lack of change in the scleral thickness as a function of refraction, all samples were analyzed following the experimental procedure described above. Figure 6 shows the values of thickness of the fibrous sclera as a function of axial length change for all the samples included in the experiment. The values ranged between 50 and 70 μm (mean value: 62 ± 6 μm). Each value corresponded to the average across the entire SHG tomography. No relationship between both parameters was found here. There was no correlation between the scleral thickness and the final refraction either.

This absence of changes with myopia was confirmed in a parallel experiment using a commercial optical coherence tomography (OCT) instrument (Spectralis, Heidelberg Engineering). Measurements provided a mean fibrous scleral thickness value of 58 ± 9 μm, with no significant difference between myopic and control eyes.

### 3.3 Organization of the scleral collagen fibers

**Figure 10:**
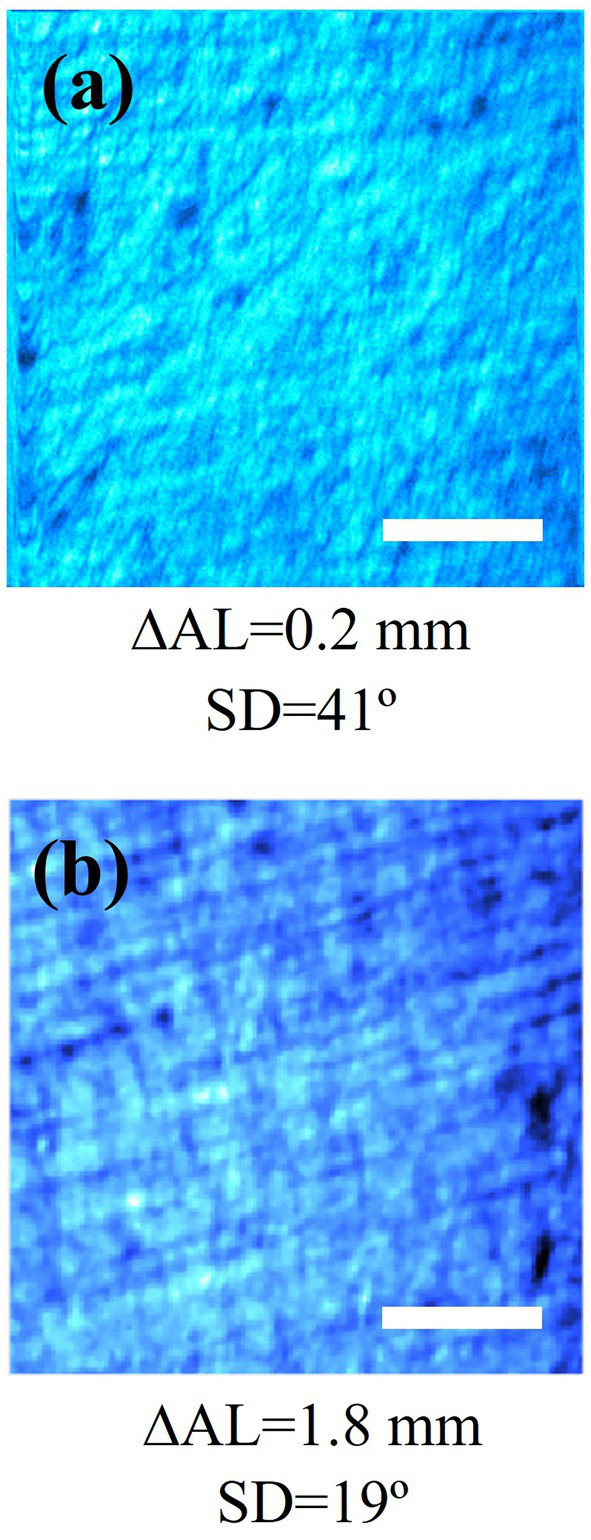
SHG images for chicken scleras with low (left) and high (right) axial length increments after deprivation. Insets indicate the change in axial length (ΔAL) and the SD values computed by the RT algorithm. Scale bar in (a,b): 50 μm.

Figure 10 shows, as representative examples, SHG images of the sclera in eyes with low (left) and high (right) axial length changes (or alternatively, low and high changes in refraction after the deprivation treatment). Directly from the images it is difficult to detect changes in the collagen structure distribution, although the scleral fibers are visible and well delineated. The insets of Figure 10 show the corresponding SD values computed through the RT procedure explained in Methods (section 2.3). Moreover, no relationship between SHG intensity and refraction or axial length was found.

Although some motion artifacts are apparent at the edges of the images, affected areas were never included in the image processing procedure. Our algorithm was designed to automatically detect and eliminate those regions from the analysis.

To further explore and quantify possible differences in the scleral collagen arrangement as a function of myopia, the SD was calculated for all samples. SD values as a function of the axial length increment are depicted in Figure 7. A decreasing (statistically significant) linear trend was observed (*R* = 0.65, *p* = 0.0004). The correlation of SD with the final axial length, not shown in the figure, was similar (SD = −16.07·AL_FINAL_ + 180.46; *R* = 0.67, *p* = 0.0002).

**Figure 11:**
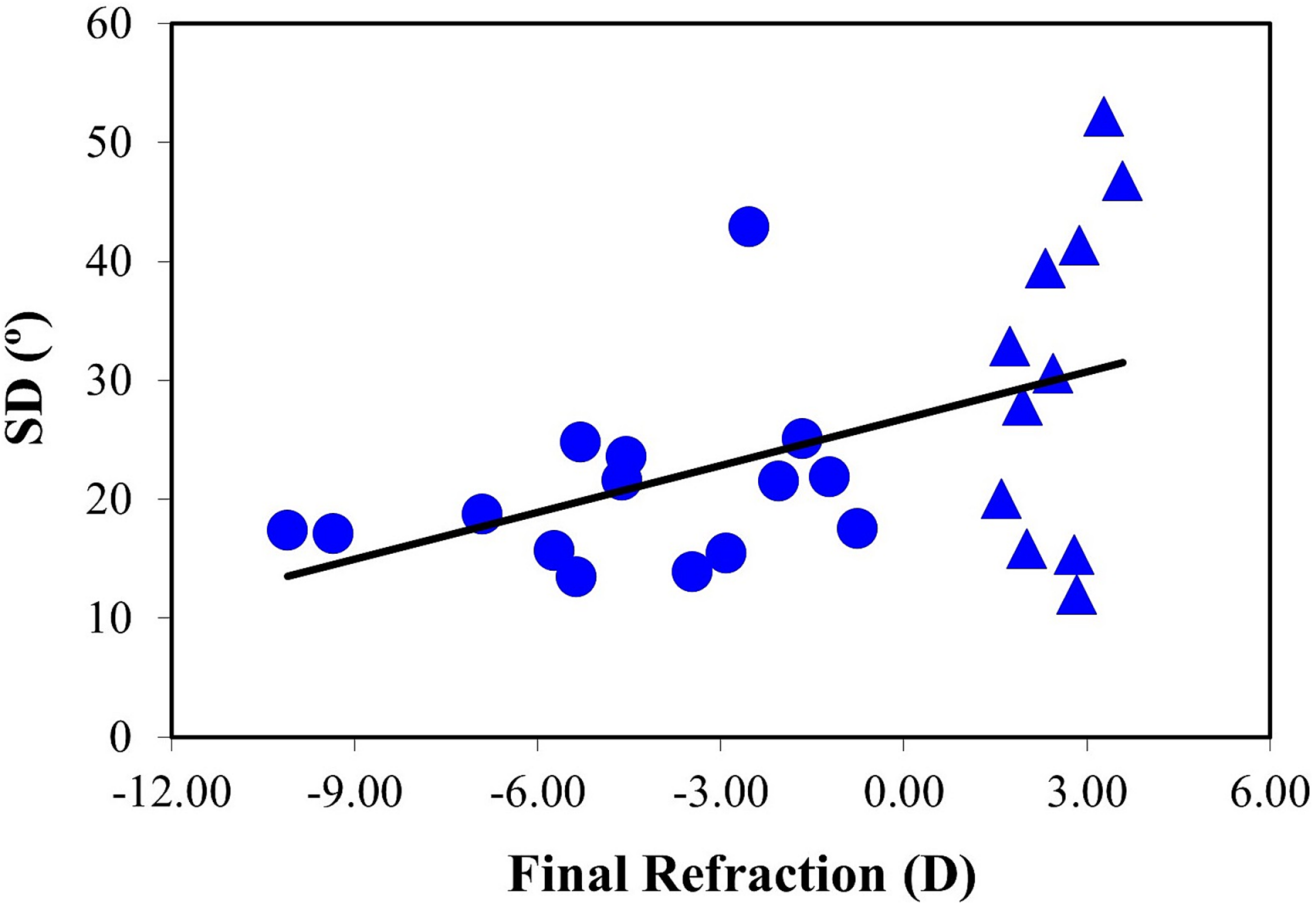
SD values as a function of the final refraction. The black line corresponds to the best linear fit (SD = 1.31·Rx_final_ + 26.80). Data for eyes with refraction values at +3D and closer to 0D are represent with a different symbol (triangles).

The relationship between SD and the final ocular refraction is shown in Figure 11 for all specimens. The fitting to a linear model shows a weaker, but still significant, correlation compared to the evolution of SD as a function of axial length increment (*R* = 0.48, *p* = 0.01). It is interesting to note a broader inter-sample dispersion in the SD values in the group of eyes with refraction at +3D and closer to 0D.

**Figure 12:**
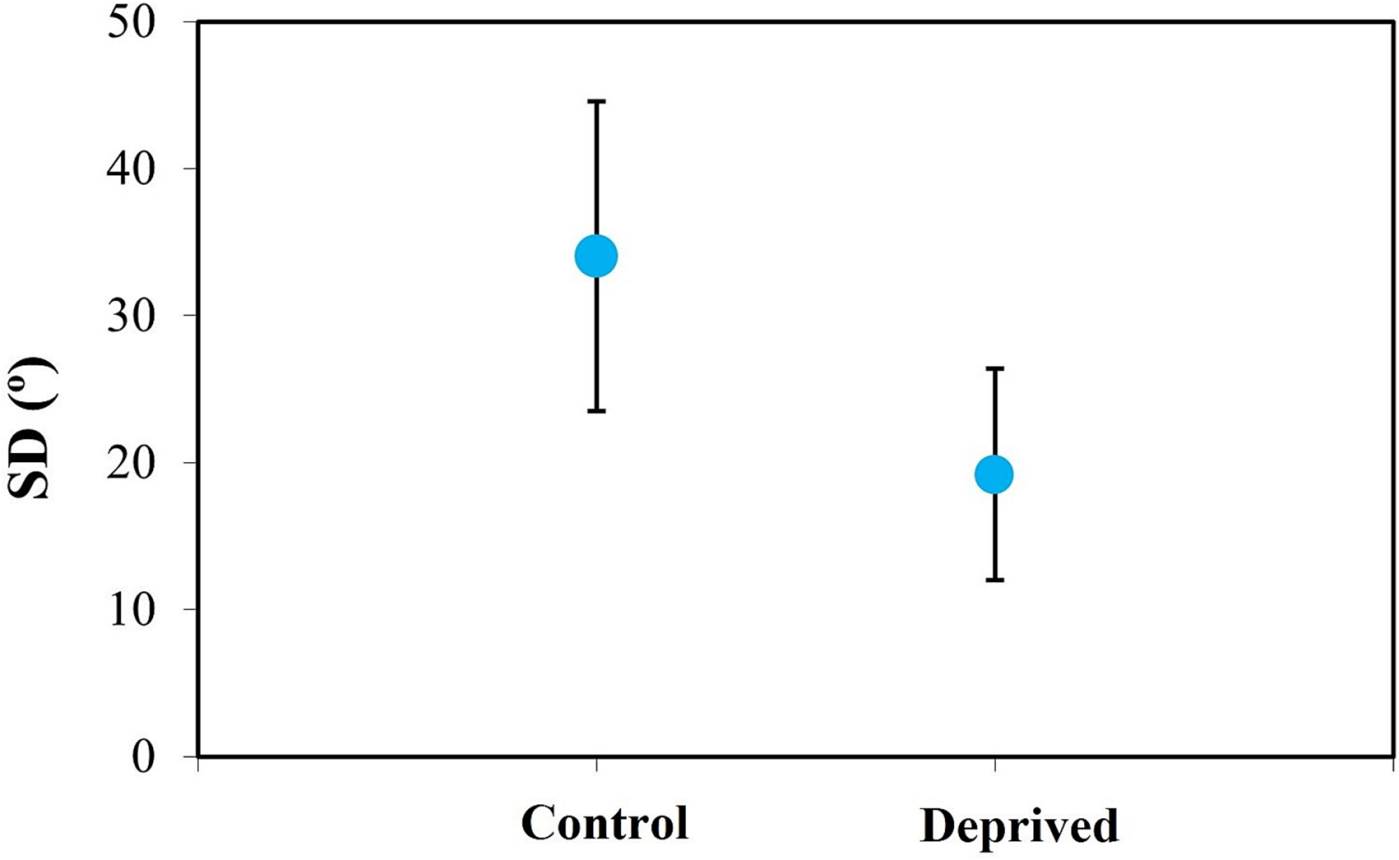
Averaged values of scleral SD in control and deprived chicken eyes (after 1 week of wearing diffuser). Error bars indicate the standard deviation.

Finally, Figure 12 depicts the overall impact of deprivation on the sclera organization of treated eyes, compared to fellow (non-deprived) eyes. While the average SD for the control group was 34 ± 11° (which is close to a non-organized collagen structure), this value was significantly reduced to 19 ± 7° in the deprived eyes (values within the range of the quasi-aligned distribution). Differences between the two groups were statistically significant (*p* = 0.02, paired *t*-test).

## 4 Discussion and conclusion

The sclera is not a static outer layer of the eye, but a dynamic tissue capable of altering the composition and structure of its extracellular matrix (i.e., collagen) in response to changes in the visual environment (38). In recent decades, there has been increasing interest in studying changes in various ocular structures due to myopia. In particular, changes in the myopic sclera as a result of axial elongation were early reported (6). As myopia progression alters the structure of this outer ocular envelope, non-invasive and quantitative analyses of the collagen fiber distribution are of great importance. These will help to better understand how myopia development alters collagen organization within the sclera.

Although morphological changes in the sclera were early recognized (2, 6), only a few studies have quantitatively characterized the extracellular matrix of the sclera and its relationship to myopia [see for instance the review by Harper and Summers (39)]. More recently, techniques such as polarization-sensitive OCT (40) and SHG microscopy (30, 31) have been used in guinea pigs for this purpose. In the former, the authors reported a positive correlation between scleral birefringence and refractive error that predicted the onset of myopia (40). On the other hand, SHG images revealed that scleral collagen fibers of form-deprivation myopic eyes were more aligned (30). Similar results were showed by Germann et al. since they found an increase in the order coefficient of the fibers when comparing the treated myopic eye and the non-myopic fellow eye (31).

In the present work we used chickens as myopia animal models. Unlike the rodent sclera (and mammals’ in general) (41) where only a fibrous layer appears, the chicken sclera consists of an inner cartilaginous layer and an outer fibrous layer that resembles mammalian sclera. Herein, SHG microscopy was used to image unstained scleral punches in both control and form-deprivation myopic chicken eyes. The thickness of the fibrous sclera was measured and the degree of organization of its collagen fibers was computed as a function of various myopia-related ocular parameters.

In contrast to the cartilaginous layer, the fibrous layer undergoes remodeling during excessive eye growth, a mechanism that might underlie the development of myopia (10). In particular, collagen fibers in the human sclera have been observed to become lamellar rather than interwoven with increasing degree of myopia (2, 6). A decrease in collagen concentration has also been observed in the posterior sclera of highly myopic humans (42). More recently, the use of wide-angle x-ray scattering mapping has shown statistically significant differences in posterior scleral fiber angle deviation when comparing non-myopic and highly myopic human eyes (>6 D) (43).

Our results on scleral remodeling in myopic eyes are consistent with previous studies in mammalian animal models and humans. The degree of organization of the scleral collagen was found to increase with myopia. In particular, there is a significant linear negative relationship between SD and the ocular axial length (slope: −16°/mm). On average, it decreased from ~50° (unorganized) to ~15° (quasi-organized). Moreover, as refraction and axial length are closely related, SD was also significantly (positively) correlated with final refraction (slope: 1.3°/D). This means that the more myopic the eye is, the higher organized the scleral collagen is.

It has been also reported that high myopia in both humans and monkeys is associated with severe thinning of the (fibrous) sclera, particularly at the posterior pole of the eye (6, 8). However, the significance of scleral thinning is still unclear. Although this might be related to a reduction in collagen fiber diameter (2), some authors claimed that this thinning was a consequence of passive stretching of the scleral tissue around the enlarged myopic eye (44, 45). However, a shrew model forced a reinterpretation of this hypothesis, since this significant thinning in the posterior part of the eye occurred after both short- and long-term deprivation treatments (9).

Our data show that the fibrous scleral thickness remains relatively constant. In our specimens, thickness values varied between 50 and 70 μm, but no correlation with the degree of myopia was observed. This result differs from previous findings in mammalian models or humans (2, 6–9, 37). Other studies have found that the thinning of the fibrous sclera in chicks is similar to what was observed in myopic mammals (16, 46, 47). On the opposite, a recent study in chicks did not find a significant difference in the thickness of the fibrous scleral layer between myopic and control eyes (48). Although this agrees with our results, there are large differences in terms of absolute fibrous sclera thickness between both experiments. Whereas Yan and colleagues measured a mean thickness of 110 μm (48), we obtained 62 μm using SHG tomography and 58 μm by means of OCT. This divergence may be partly explained by the fact that chicken hybrid line in Yan’s study was different from ours. Moreover, their animals were also slightly older.

Additional physical parameters such as the fiber size or the tissue elasticity were out of the scope of this work. However, it is interesting to note that smaller collagen fiber diameters in the fibrous sclera of chicken myopic eyes (16, 49) and mammalian models (including humans) (2, 9, 30) have been previously described. This narrowing of the fibers may be associated with both biochemical and biomechanical changes in the scleral extracellular matrix (15, 42, 50). Scleral elasticity has been showed to increase in eyes developing myopia, mainly due to the reduced collagen content (51). Other evidence suggests that the biomechanical properties of sclera (elasticity and creep) may play a significant regulatory role in the axial elongation of myopic eyes (15, 52). Whereas elasticity is related to the immediate change in the tissue length when a force is applied (i.e., extension vs. load), creep describes the slow, time-dependent extension/compression under a constant load (i.e., extension vs. time) (15). Although biomechanical parameters have not been specifically addressed here, further analyses on these may help to clarify whether scleral collagen changes in myopia result from passive stretch or from active tissue remodeling.

In conclusion, SHG microscopy images of the chicken scleral tissue were used to objectively study the changes produced during the development of deprivation myopia. The spatially resolved distribution of scleral collagen fibers was visualized and quantified as a function of ocular refraction. The axial elongation associated with increasing amounts of myopia is closely related to the rearranging of the scleral tissue. Our experiment showed a process of remodeling of the posterior sclera during axial elongation. The collagen pattern changes from a non-organized distribution into a quasi-organize arrangement.

## Data Availability

Data underlying the results presented in this paper may be obtained from the authors upon reasonable request.
